# Outcome measures for assessing change over time in studies of symptomatic children with hypermobility: a systematic review

**DOI:** 10.1186/s12887-021-03009-z

**Published:** 2021-11-29

**Authors:** Muhammad Maarj, Andrea Coda, Louise Tofts, Cylie Williams, Derek Santos, Verity Pacey

**Affiliations:** 1 Narrabeen Sports Medicine Centre, Sydney Academy of Sport, Sydney, Australia; 2 Department of Health Sciences, Newcastle University, Newcastle, Australia; 3 Priority Research Centre Health Behaviour, Hunter Medical Research Institute HMRI, Newcastle, Australia; 4 Department of Health Professions, Macquarie University, Sydney, Australia; 5 Department of Physiotherapy, Monash University, Melbourne, Australia; 6 Department of Health Sciences, Queen Margaret University, Scotland, UK

**Keywords:** Fatigue, Outcome measures, Paediatrics, Hypermobility, Pain, Function, Quality of life

## Abstract

**Background:**

Generalised joint hypermobility (GJH) is highly prevalent among children and associated with symptoms in a fifth with the condition. This study aimed to synthesise outcome measures in interventional or prospective longitudinal studies of children with GJH and associated lower limb symptoms.

**Methods:**

Electronic searches of Medline, CINAHL and Embase databases from inception to 16th March 2020 were performed for studies of children with GJH and symptoms between 5 and 18 years reporting repeated outcome measures collected at least 4 weeks apart. Methodological quality of eligible studies were described using the Downs and Black checklist.

**Results:**

Six studies comprising of five interventional, and one prospective observational study (total of 388 children) met the inclusion criteria. Interventional study durations were between 2 and 3 months, with up to 10 months post-intervention follow-up, while the observational study spanned 3 years. Three main constructs of pain, function and quality of life were reported as primary outcome measures using 20 different instruments. All but one measure was validated in paediatric populations, but not specifically for children with GJH and symptoms. One study assessed fatigue, reporting disabling fatigue to be associated with higher pain intensity.

**Conclusions:**

There were no agreed sets of outcome measures used for children with GJH and symptoms. The standardisation of assessment tools across paediatric clinical trials is needed. Four constructs of pain, function, quality of life and fatigue are recommended to be included with agreed upon, validated, objective tools.

## Background

Children with generalised joint hypermobility (GJH) and associated symptoms have been described within the literature under multiple diagnostic labels which have differed over time. Generalised joint hypermobility (GJH) describes abnormally high joint ranges of movement in multiple joints [1] with approximately one-fifth of children with GJH reporting symptoms [2, 3]. Currently used diagnostic labels describing children with GJH with associated symptoms include Generalised Hypermobility Spectrum Disorder (G-HSD) [4], and hypermobile Ehlers-Danlos Syndrome (hEDS), which further incorporates an extended phenotype including skin involvement, tissue fragility or a marfanoid body habitus [5]. These conditions were previously referred to as Joint Hypermobility Syndrome (JHS) or EDS-Hypermobile type, with experts previously reporting a lack of clinical distinction between the two [6, 7]. The term “children with GJH and associated symptoms” will be used throughout this review to indicate any of the current or previously used terminology for this condition.

Children with GJH and associated symptoms report chronic pain [8], fatigue [9] and functional difficulties [10] that have a negative impact on their quality of life [11, 12]. Chronic joint pain is often exacerbated following physical activity [13] with lower limb pain being the most common location described [14]. Joint instability episodes and frequent soft tissue injuries have also been reported [14]. Functional difficulties reported include motor development challenges [15], muscle torque deficits and poor proprioception [16] resulting in a negative influence on school and/or social activity participation [17]. Some children with GJH also describe systemic symptoms including orthostatic intolerance, functional gastrointestinal disorders and stress incontinence [11, 14], with a greater number of systemic symptoms leads to worse functional disability [18]. Additional psychological symptoms may also result in poorer quality of life than typically developing children [12, 14, 19, 20].

Validated, reliable outcome measures enhance our understanding of the natural history of a condition and aid evaluation of treatment effectiveness. Despite the importance of such validated outcome measures in paediatric populations [21] there are no condition specific outcome measurement instruments for children with GJH and associated symptoms. Consequently, the natural history of the condition is poorly understood, and recent systematic reviews and meta-analyses have been largely inconclusive, partially due to the lack of standardised outcome measures used between studies [22–25]. Identification of outcomes reported in the literature to monitor change in children with GJH and associated symptoms informs rigorous methodology incorporating expert researcher and health professionals consensus, in conjunction with patient and family involvement, to develop a minimum core outcome set for research reporting [26]. Therefore, this study aimed to synthesise outcome measure type and use in interventional or prospective longitudinal studies of children with GJH and associated symptoms.

## Methods

This systematic review was performed according to the Preferred Reporting Items for Systematic Reviews and Meta-Analyses (PRISMA) guidelines [27]. The protocol was registered on the Prospective Register of Systematic Reviews (PROSPERO) database (registration number CRD 42,018,081,835) prior to commencement of database searches.

### Definition of Beighton Score

The 9-point Beighton score (BS) is a dichotomised standardised method [28, 29] widely used for assessment of GJH and associated symptoms as a clinical diagnostic tool as well as in hypermobility related research studies [30, 31]. The Beighton score typically includes four or more positive finding for both upper and lower joints as follows: passive dorsiflexion of elbows, knees and fifth finger beyond 90° angle; forward flexion of trunk with full extension of knees and hands resting flat on the floor; and passive opposition of each thumb to the forearm flexor surface [28].

Although the cut-off of ≥ 4/9 is the most commonly used BS, however this score is largely influenced by age, gender and ethnicity [32–34]. Therefore, the new 2017 International Classification of the Ehlers-Danlos syndromes has recommended the use of age specific cut-offs of ≥ 4/9 (adults older than 50 years), ≥ 5/9 (pubertal children and adults 50 years or lower) or ≥ 6/9 (prepubertal children) for BS [5]. Since BS was originally not intended as a diagnostic tool [28] it has not been directly validated to detect GJH in adults. However BS has been found to be a valid method in assessing hypermobility in children with GJH [32].

### Search Strategy

Medline (via PubMed), CINAHL and Embase databases were searched from inception to 16th March 2020 using the terms and strategy presented in Table 1. Further studies were retrieved from backward manual searches of references lists of included studies. There was no restriction imposed by publication year or language.

**Table 1:** Search terms and search strategy documentation for PubMed^a^

1. Paediatric* OR Pediatric*	
2. Child* OR Juvenile* OR Adolescent*	
3. #1 OR #2	
4. Measure* OR Therap* OR Outcome* OR Hypermob*	
5. #3 AND #4	
6. Elhers* OR Double-Join* OR Brighton OR Beighton	
7. # 5 AND #6	

^Notes.*=truncate^

^a^This search strategy was modified for CINAHL and Embase databases

### Eligibility criteria

Randomised controlled trials (RCTs), quasi-RCTs, longitudinal and cohort studies were included. The study populations were restricted to children and adolescents aged between 5 and 18 years, diagnosed with GJH, as defined by the authors of the studies, and associated lower limb symptoms. Given the considerable heterogeneity of cut-offs for Beighton score reported in literature its limitation as a clinical diagnostic tool [30, 31], we included all relevant studies that assessed children with GIH and associated symptoms. Included studies were required to describe outcome measures utilised at least 4 weeks apart in order to identify change over time.

Studies focusing on upper limb only outcome measures, or studies including children with other hereditary connective tissue disorders or syndromic conditions associated with GJH, were not included.

### Study selection

Titles, abstracts and full-text article screening was performed independently by two authors (MM and AC) against the inclusion/exclusion criteria. Any discrepancies were resolved either by discussion between the two reviewers or by a third author (DS) until consensus was reached.

### Data extraction

Two reviewers (MM and CW) independently extracted relevant data from included full text articles. Data extraction was performed on a standardised template and included: the primary author of the study, year of publication, country, study design, participant demographics (sample size, gender and age), intervention characteristics (type, duration and follow-up) where applicable, and outcome measures used to assess change. Any unresolved disagreements were mediated by the remaining authors (AC, LT, DS and VP).

### Risk of bias assessment

The methodological quality of all eligible studies was reviewed independently by two authors (MM and DS) using the Downs and Black checklist. Any disagreements were discussed until a consensus was reached or resolved by a third author (AC). The Downs and Black checklist [35] is a validated methodological quality assessment tool covering 5 domains of reporting, external quality, internal validity (bias), internal validity confounding or selection bias, and statistical power [36].

#### Data analysis

Descriptive statistics were used to characterise the included studies participant population, duration and intervention. Outcome measures used were categorised into patient- or parent-reported (PRO) or clinician-reported (CRO) outcomes, and the broad constructs which were being assessed. The frequency of individual outcome measures used to assess each construct was then tallied. A narrative synthesis of the outcome measures used across study type and participant age was performed, including presentation of the baseline scores on measures. To provide a description of the change over time, the mean change, and variance in this, was also presented. Where 95% CIs were not presented to represent the variance in change, they were calculated.

## Results

### Selection strategy and methodological appraisal

**Fig. 1:**
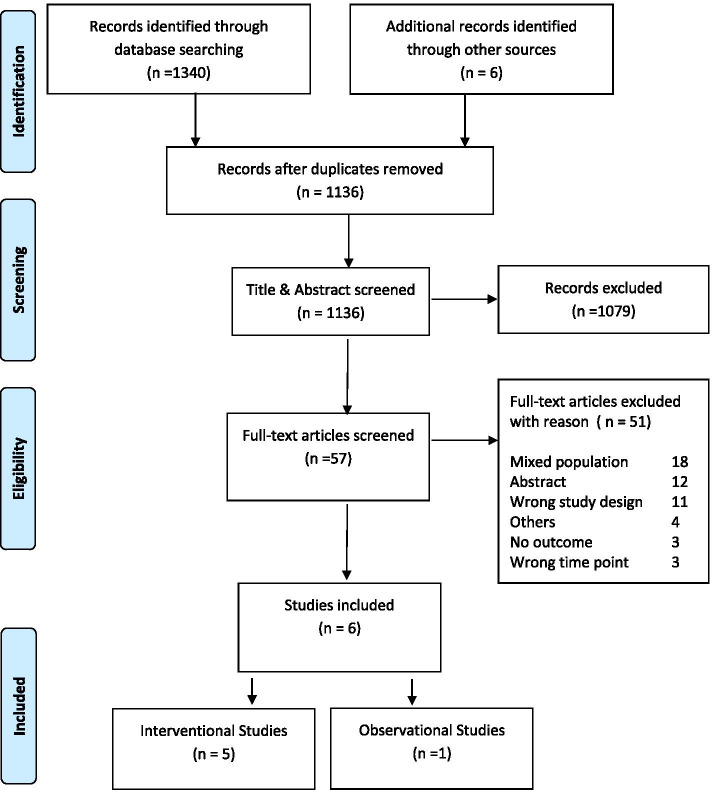
Flow diagram of the study

From a total of 1136 articles identified through the searches, 57 articles were deemed eligible for full-text screening with six studies eligible to be included in this review (Fig. 1). Five interventional studies were identified, these were four RCTs and one pre-post cohort study. The sixth was a prospective observational study. All included studies were published during the last ten years.

The methodological quality of the six studies was described in Table 2. Main limitations of the studies included poor description of principal confounders, lack of participant blinding, not reporting adverse events related to intervention(s), and not minimising bias for data collection. The strength of included studies were clearly described main outcomes, recruitment of participants from the same target population as well as the use of validated and reliable outcome measures appropriate for the general paediatric population. While all interventional studies clearly described the trial and control interventions, only one study blinded participants to the interventions while the other four studies demonstrated blinding of assessors to the group allocation of intervention or controls.

**Table 2:** Assessment of methodological quality of eligible studies using Downs & Black checklist (Downs and Black 1998)^a^

Items	Criteria	Bale (2019)	Hsieh (2018)	Revivo (2018)	Pacey (2013)	Kemp (2010)	Scheper (2017)
REPORTING							
**1**	Study hypothesis/aim/objective clearly described	1	1	1	1	1	0
**2**	Main outcomes in Introduction or Methods section	1	1	1	1	1	1
**3**	Patient characteristics clearly described	1	1	1	1	1	1
**4**	Relevant interventions including controls clearly described	1	1	1	1	1	NA
**5**	Distributions of principal confounders clearly described	0	0	1	0	0	2
**6**	Main findings (including outcomes) clearly described	1	1	1	1	1	1
**7**	Estimates of random variability in data for the main outcomes provided	1	1	1	1	1	1
**8**	All important adverse events related to intervention(s) reported	0	0	0	1	0	NA
**9**	Patient characteristics lost to follow-up described	1	1	1	1	0	0
**10**	Actual probability values for main outcomes reported	1	1	1	1	1	1
EXTERNAL VALIDITY							
**11**	Subjects asked to participate were representative of target populations	1	1	1	1	1	1
**12**	Subjects prepared to participate were representative of target populations	1	1	1	1	1	1
**13**	Treatment facilities and delivery were representative of target populations	1	1	1	1	1	1
INTERNAL VALIDITY – bias							
**14**	Study participants blinded to intervention administered	0	0	0	1	0	NA
**15**	Investigators blinded to assessment of main intervention outcomes	1	1	0	1	1	NA
**16**	Any data dredging was made clear at onset of study	0	0	1	1	1	0
**17**	Analyses adjust for different lengths of follow-up of participants	1	0	1	1	0	1
**18**	Statistical tests to assess the main outcomes were appropriate	1	1	1	1	1	1
**19**	Reliability of compliance with intervention(s)	1	1	1	1	0	NA
**20**	Main outcome measures used accurate in terms of validity and reliability.	1	1	1	1	1	1
INTERNAL VALIDITY - confounding (selection bias)							
**21**	All participants were recruited from the same target population	1	1	1	1	1	1
**22**	All participants were recruited over the same period of time	1	1	0	1	1	1
**23**	Participants were randomised to intervention group(s)	1	1	0	1	1	NA
**24**	Randomised intervention assignment was concealed from both participants and investigators	0	0	0	1	0	NA
**25**	Adequate adjustment for confounding	0	0	0	0	0	1
**26**	Lost to follow-up considered	1	0	1	1	0	0
**27**	Statistical power- clinical meaningful effect or power calculation reported ^b^	1^~^	1	1	1	1	1

^~^Power calculation reported but not clinically meaningful

^a^ The scoring given for each criteria was 1 point for ‘Yes’ or 0 point for ‘No’ except question 5 which is scored as 2 for ‘Yes’, 1 for partially or 0 for ‘No’ related to the distribution of principle confounders [35]. For observational study NA=Not applicable.

^b^ Only one point was awarded to an interventional study powered to detect a meaningful clinical effect [37, 38]

### Characteristics of the eligible studies

The main characteristics of included studies are summarised in Table 3. There were 388 participants in total from the six studies. Overall, studies included primarily female participants, and ranged in duration from 2 months to 3 years. Interventions included either exercise therapy alone (n = 3) or combined with orthotics (n = 1) or multidisciplinary care (n = 2). All participants were recruited from children’s hospital clinics.

**Table 3:** Characteristics of eligible studies included in this systematic review

Study (year) Country	Study Design	Participant characteristics					Outcome assessment		
		Participants (n)	Drop out (%)	Age in years Mean (SD) % Female	Beighton score^a^ Mean (SD)	Recruitment site	Treatment or intervention group	Control group	Duration (Follow-up ^b^)
Bale et al. (2019) [39] UK	Randomised controlled trial	119 baseline 111 At 3months 105 At 12 months	7% 12%	9.4 (3.2) 55%	5.7 (1.4)	Children’s department at tertiary Hospital	therapy intervention (Tertiary PT and OT x5 sessions)	Standard care (medical assessment and allied health referrals)	2 months (1, 10 months)
Hsieh et al. (2018) [40] Taiwan	Randomised controlled trial	52 Baseline 50 At 3 months	4%	6.6 (0.6) 46%	7.5 (1.6)	Outpatient rehabilitation center – teaching hospital	Physical therapy & orthotics with customised insoles	Physical therapy & podiatry without customised insoles	3 months
Kemp et al. (2010) [41] UK	Randomised controlled trial	57 Baseline 32 At 3 months	44%	10.9 (2.5) 33%	5.8 (1.6)	Rheumatology Outpatient department	Psychosocial & physical therapy targeted to improve functional stability of symptomatic joints	Generalised therapy to improve muscle strength & fitness	2 months (3months follow-up)
Pacey et a1. (2013) [42] Australia	Randomised controlled trial	29 Baseline 26 randomised 25 2 months	14%	12.1(2.9) 66%	7.1 (1.2)	Physiotherapy department in a teaching hospital	Physical therapy: Muscle strength & motion control performed into full range of knee hyperextension	Physical therapy: Muscle strength & motion control performedinto knee extension neutral range	2 week baseline without treatment followed by 8 treatment sessions and home exercises over 2 months
Revivo et al. (2019) [43] UK	Pre-Post retrospective	30 Baseline 26 2 months	13%	14.0 (2.8) 90%	>4	Hospital Outpatient multidisciplinary pain management clinic	Physical therapy, occupational therapy, psychology counselling, & weekly paediatric rehabilitation follow-up	None	1.5-2 months
Scheper et al. (2017) [44] Australia	Observational longitudinal	101 Baseline 81 3 years	20%	11.5 ± 3.1 55%	7 ± 1.6	Tertiary hospital Outpatients clinics	No restrictions on treatment of participants	None	3 years

Abbreviations. GP: Generalised Physiotherapy; HTG : Hypermobility treatment group; NTG: Neutral treatment group; TP: Targeted Physiotherapy. PT = physiotherapy OT= occupational therapy

^a^ Based on a 9 point scale [28]. The score is combined for both treated and control

^b^ Follow-up is post-intervention

### Outcome measures

Table 4 provides descriptions of the outcome measures and instruments used in the studies where the change in these measures over time was able to be collected or provided by the authors. There were 20 distinct outcome instruments measuring the four constructs of pain [39–42, 44], function [39–44], quality of life [39–42, 44] and fatigue [44] which included 15 PROs (7 patient-reported and 8 parent-reported) and 4 CROs. All PRO instruments except one (PGIC: Patient’s Global Impression of Change) [42] have been validated for use in the paediatric population. Pain was the most common construct measured, using 4 different PROs [39, 41–44], the patient-reported Visual Analogue Scale (VAS) [39, 41, 42, 44], parent-reported VAS [39, 41], Numerical Rating Scale (NRS) [43], and the Wong-Baker Faces Pain Scale (WBFPS) [39].

**Table 4:** Outcome measures categorised according to pain, function and quality of life

Outcome measures			Follow-up Timeframe ^d^	Baseline Mean (SD)	Mean change in outcome at follow-up ^a^	95% CI
Scale	Test details	Type				
PAIN (Intensity)						
VAS [45, 46] (Visual Analogue scale)	0-100 0 = no pain 100 = worst pain	PRO	2 months [42]	Neutral treatment group: 40.0 (16.6)	-19.9	NR
				Hypermobility treatment group: 38.6 (16.9)	-9.19	NR
				Combined groups: 39.4 (14.2)	-14.5	-5.2, -23.8
			5 months ^e^ [41]	Targeted Physiotherapy: 55.5 (21.3)	-21.2	-38, -4.5
				General Physiotherapy: 62.1 (24.1)	-30.6	-50.16, -11.0
				Combined groups: 57.6 (20.1)	-25.8	-38.5, -13.1
WBFPS [47, 48] (Wong-baker faces pain scale)	0-5 0 = no pain 5 = worst pain	PRO	12 months [39]	Intervention: 2.2 (1.4)	-1.6	-2.1, -1.1
				Control: 2.5 (1.6)	-1.6	-2.0, -1.2
PAIN (Intensity)						
VAS-P [49] (Visual Analogue scale-Parental)	0-100 0 = no pain 100 = worst pain	PRO ^b^	5 months [41]	Targeted Physiotherapy: 45.1 (23.0)	-21.6	-33.2, -10.0
				General Physiotherapy: 48.4 (22.9)	-12.	-23.3, 0.9
				Combined groups: 46.7 (22.7)	-17.2	-25.3, -9.1
			12 months [39]	Intervention: 33.8 (24.8)	-6.8	-14.3, 0.7
				Control: 40.6 (27.5)	-7.3	-15.4, 0.8
FUNCTION						
CHAQ [50] (Childhood Health Assessment Questionnaire)	0-3 0 = Without any difficulty 1 = With some difficulty 2 = With much difficulty 3 = Unable to do	PRO ^b^	2 months [42]	Neutral treatment group: -0.13 (0.44)	0.12	NR
				Hypermobility treatment group: 0.04 (0.71)	0.02	NR
				Combined groups: -0.5 (0.6)	0.07	-0.1, 0.2
			5 months [41]	Targeted Physiotherapy: 0.62 (0.65)	-0.15	-0.3, -0.02
				General Physiotherapy: 0.76 (0.68)	-0.16 (	-0.4, 0.1
				Combined groups: 0.69 (0.66)	-0.15	-0.3, -0.02
			12 months [39]	Intervention: 0.84 (0.62)	0.04	0.1, 0.2
				Control: 0.86 (0.72)	−0.02	-0.12, 0.08
Dynamometry [51] Measurement of strength	Grip strength. Units: kilopascals	CRO	12 months [39]	Intervention: 57.0 (25.0)	4.7	0.1, 9.3
				Control: 59.4 (31.7)	7.3	2.9, 11.7
	Knee flexor and extensor strength. Units: Newtons		2 months [42]	Neutral treatment group: 4.0 (1.7)	0.88	NR
				Hypermobility treatment group: 4.4 (2.4)	1.21	NR
				Combined groups: 4.2 (2.0)	1.1	0.4, 1.7
FUNCTION						
M-ABC2 [52] (Movement Assessment Battery for Children, 2nd Edition)	Measures coordination Raw scores converted to centiles (0 – 100) with higher centiles indicating better performance compared to peers	CRO	12 months [39]	Intervention: 33.4 (26.7)	3.8	-1.7, 9.3
				Control: 35.6 (30.1)	10.8	5.4, 16.2
PODCI [53, 54] (Pediatric Outcomes Data Collection Instrument)	0-100 100= highest functioning	PRO ^b^	3 months [40]	Transfer and basic mobility domain Intervention: 82.1 (14.8)	11.8	0.30, 1.43
				Control: 94.2 (14.8)	1.2	-0.46, 0.62
6 MWT [55] (6 min walk test)	Maximum distance walked in 6 minutes (meters/leg length)	CRO	36 months [44]	Able/moderate: 7.3 (1.6) ^c^	-1.5	-1.3, -4.5
				Severe: 5.3 (1.6)	-2.3	-2.4, -2.7
No. of flights of stairs climbed in 2 min [56]	Assesses functional ability in stairclimbing	CRO	2 months [42]	Neutral treatment group: 16.3 (5.0)	3.8	NR
				Hypermobility treatment group: 20.9 (6.7)	-0.33	NR
				Combined groups: 18.6 (5.7)	1.7	-0.5, 3.9
Quality of Life						
CHU9D [57] (Child Health Utility 9D)	0-4 higher scores indicate poorer HRQoL	PRO	12 months [39]	Intervention: 0.85 (0.10)	0.02 (0.09)	-0.004, 0.04
				Control: 0.85 (0.12)	0.00 (0.12)	-0.03, 0.03
CHQ-PF50 [58] (Child Health Questionnaire)	0-100 0 = worst QoL 100 = Best QoL	PRO ^b^	2 months [42]	*Physical summary score*		
				Neutral treatment group: 32.0 (11.9)	10.1	NR
				Hypermobility treatment group: 41.6 (15.0)	2.3	NR
				Combined groups: 38.0 (12.6)	5.3	1.7, 8.9
				*Psychological summary score*		
				Neutral treatment group: 46.4 (12.3)	-0.9	NR
				Hypermobility treatment group: 46.3 (9.0)	8.1	NR
				Combined groups: 48 (10.3)	2.7	-0.3, 5.8
Quality of Life						
PGIC [59, 60] (Patient global impression of change)	1-7 1= very much improved 7= very much worse	PRO	2 months [42]	Neutral treatment group: 0.3 (1.1)	1.4	NR
				Hypermobility treatment group: 0.2 (0.9)	1.6	NR
				Combined groups: 0.2 (1.0)	1.5	1.0, 2.0
PODCI [53] (Pediatric Outcomes Data Collection Instrument) Pain comfort Happiness	0-100 Higher score means higher health-related quality	PRO ^b^	3 months [40]	**1. Pain/comfort:**		
				Intervention: 83.9 (16.2)	4.9	-0.22, 0.87
				Control: 84.4 (17)	-1.2	-0.61, 0.47
				**2. Happiness:**		
				Intervention: 79.5 (18.7)	-0.2	-0.55, 0.53
				Control: 80.7 (15.9)	-0.9	-0.60, 0.48
PedsQL parent proxy-reported format [61] and [62] (Pediatric Quality of Life Inventory-Generic Core Scale)	0-100 Lower score indicates lower quality of life	PRO ^b^	3 months [40]	**1. Physical**		
				Intervention: 62.3 (19.9)	3.9	-0.35, 0.73
				Control: 79.2 (20.1)	-8.1	-0.95, 0.14
				**2. Psychosocial**		
				Intervention: 65.6 (16.3)	0.9	-0.49, 0.59
				Control: 73.8 (18.8)	0.3	-0.52, 0.56
Quality of Life						
Global-VAS (parent’s global assessment)	0-100 0 = no impact of hypermobility 100 = high impact of hypermobility	PRO ^b^	5 months [41]	Targeted Physiotherapy: 36.1 (26.4) General	-17.6	-31.1, -4.1
				Physiotherapy: 37.2 (25.3)	3.7	-7.8, 15.3
				Combined groups: 36.6 (25.7) n = 32	-7.6	-17.2, -2.0

Abbreviations. 95% CI: 95% Confidence Interval; CRO: Clinician-reported outcome; GP: Generalised Physiotherapy; HRQOL=health-related quality of life; QoL: quality of life; PRO: Patient-reported outcome

^a^ difference in change score from baseline (outcome-baseline)

^b^ Indicates Parent reported outcomes

^c^ Data calculated by primary author to demonstrate the difference between children of different severity and supplied to the authorship team upon request. This was only able to be provided for 6MWT, not the other variables

^d^ Time points are when outcome measurements are reported

^e^ Authors converted the faces pain scale to a 0–100 scale to combine with VAS data

When considering all the PROs used, the patient-reported VAS [39, 41, 42, 44], Childhood Health Assessment Questionnaire (CHAQ) [39, 41, 42, 44] and parent-reported VAS [39, 41] were the only PRO measures used in more than one study.

Function was assessed with a total of nine different assessment tools. Five PROs were used to assess function including the CHAQ [39, 41, 42], Pediatric Outcomes Data Collection Instrument (PODCI) [40], and the Bath Adolescent Pain questionnaire (BAPQ 61) [43]. The Bath Adolescent Pain Parent Impact Questionnaire (BAP-PIQ) was also used to assess the impact of the child’s condition on the parents daily function [43], and the Adolescent Physical Activity Recall Questionnaire (APARQ) scale to assess a child’s physical activity [44]. The 4 CROs used to assess function included the 6 min walking test to assess walking endurance [44], the ability to climb stairs in a set time [42], the Movement Assessment Battery for Children, 2nd Edition (M-ABC2) [39] to assess gross motor skills, and muscle strength [39, 42]. Strength was measured in two studies, however they each assessed different muscle groups [39, 42].

Quality of life was described using the three different patient-reported outcome scales; Child Health Utility 9D (CHU9D) [39], PGIC [42] and Pediatric Quality of life questionnaire (PedsQL) [44]. The change in the child’s quality of life reported by parents was measured using PODCI [40], Child Health Questionnaire (CHQ-PF50) [42], PedsQL parent proxy-reported format [40], and Global-VAS (parent’s global assessment) [41]. Only one study measured fatigue, using the PedsQL- Multi-dimensional Fatigue Scale [44].

## Discussion

There was significant heterogeneity in the use of instruments across studies included within this systematic review. Multiple studies measured pain intensity, function and quality of life constructs; however fatigue was measured in only one study, which found it to be an independent predictor of functional deterioration. All measures used demonstrated change over time.

The identified PRO measures used similar item sets without taking into account lifestyle or severity of the condition. This limits their translational capabilities into clinical practice. Despite the advantage of assessing the same outcome repeatedly in a clinical trial for research, measuring changes in symptoms tailored to the child’s individual presentation may be more beneficial to inform clinical decisions [63]. Children with GJH and associated symptoms commonly describe variable symptoms depending on their lifestyles, environmental condition or individual characteristics [64]. The use of PROs with more inclusive questions that capture all relevant domains to an individual and their specific condition may provide a more useful alternative to better assist clinicians translate evidence into practice. Furthermore, the use of measures specifically validated for children with GJH and associated symptoms, would provide a clearer understanding of the natural change in symptomatology of children with GJH and associated symptoms, and more robust evidence for the effectiveness of interventions in this patient population.

Therapy aims to improve quality of life and reduce disability in children with GJH and associated symptoms [65]. It is unknown if generic outcome measures alone would enable reporting with adequate validity and sensitivity [66, 67]. In this present review, the majority of studies administered multiple instruments, combining both PRO and CRO scales. Further evaluation with qualitative methodology may provide valuable insight into the priorities and needs of children with GJH and associated symptoms, and their caregivers. This may refine the constructs and specific outcome measures used in future research and clinical practice.

Studies of intervention effectiveness and/or change with time in well described and defined populations with symptomatic generalised hypermobility using well validated robust measures that can be used in both the research and clinical contexts will support easier interpretation and comparison across both contexts. Each individual study provides valuable additional original information, but analysis of multiple studies will provide a higher level of evidence in the future This allows for comparisons between settings, interventions and patient groups to get a broader understanding of the measures used and provide meaningful informed assessment of therapies. Lack of standardisation, together with the limited number of interventional or prospective cohort studies, has hampered quantitative synthesis of efficacy of interventions using meta-analysis in previous systematic reviews (23,24). In other paediatric rheumatological health conditions, such as Juvenile Idiopathic Arthritis (JIA), established and revised core sets of outcomes determined through expert health professional consensus [68, 69] have been used. In line with the findings of our review, the JIA international workgroup prioritised pain, function and quality of life (overall wellbeing) as mandatory domains for research. In addition, fatigue prioritised by patient/parents was considered an important construct outcome measure for inclusion in the most recent update [69].

There is a substantial impact of fatigue on quality of life of children with GJH and associated symptoms [12, 14, 18, 19, 70]. The most poorly functioning children diagnosed with hypermobility and associated symptoms experience worse fatigue and higher pain intensity than their peers [44]. No single assessment instrument has been identified to measure the severity of fatigue and its impact on wellbeing in this population group. Given the significance of fatigue, strong consideration of fatigue measurement is recommended within a core set of outcome measures.

Studies have also reported children and parents describing systemic symptoms such as gastrointestinal involvement and stress incontinence associated with poorer quality of life relating to hypermobility [14, 71, 72]. Outcome measures measure that identify the impact of different systemic symptoms on child function and quality of life may also be useful to guide clinical management and assess the efficacy of interventions in this population.

This review was strengthened through the registration of a protocol, adherence to established PRISMA guidelines, and appraisal of methodological quality using a tool with substantial inter-rater reliability [73], and one that highlighted for use in assessing the quality of non-randomised controlled studies [74]. We acknowledge a number of limitations to this review. The research strategy used within this review only identified studies published in English despite no language restrictions placed on eligibility criteria. This study also focused on outcome measures for children with GJH and associated lower limb symptoms and did not assess outcome measures relevant to children’s other symptoms affecting the upper limb and spine, or other multisystemic features. While limiting the review, lower limb symptoms were chosen as they are consistently reported most frequently in this population [75]. Additionally, it was not the aim of the review to assess the validity or reliability of the included measures in the paediatric or condition-specific population.

We are also not able to comment on the association between degree of joint hypermobility and the outcomes of pain, fatigue, quality of life and function since there is no available clinical diagnostic markers for hypermobility disorders or tools to assess such relationship. The application of BS as a dichotomise measure can only provide information on presence of hypermobile joint [4, 5, 31]. Furthermore, there are currently no gold standard method for GJH diagnosis to allow measurements of sensitivity and specificity of the BS as a diagnostic tool and therefore it limits BS application beyond an initial screening tool [30]. As the overarching aim of our systematic review was to collect evidence on the outcome measures related to symptomatic hypermobility and therefore determining the relationship between degree of hypermobility and these outcomes was outside the scope of our review. As far as we are aware there are no studies that have correlated grade of lower limb hypermobility to the degree of these domains in children and certainly this is a valid question worth exploration in future studies.

## Conclusions

An agreed set of core outcome measures for children with GJH and associated symptoms is warranted. More precisely defined diagnostic criteria for children with hypermobility related disorders, in conjunction with standardised reporting of the effectiveness of interventions using similar outcome measures in future studies will produce better quality evidence to facilitate translation into healthcare services. We recommend the development of a core set of outcome measures based around the four constructs of pain, function, quality of life and fatigue. Mixed methodology, including the views of children living with GJH and associated symptoms and their families on what is important to them, combined with expert consensus, validation of generic outcome measures in this population and development of condition specific outcome measures, would provide the ideal final core outcome set for future use.

## Data Availability

The datasets used and/or analysed during the current study are available from the corresponding author on reasonable request.

## Abbreviations

APARQ: Adolescent Physical Activity Recall Questionnaire BAP-PIQ:Bath Adolescent Pain Parent Impact
BAPQ 61: Bath Adolescent Pain questionnaire
CHAQ: Childhood Health Assessment Questionnaire
CHQ-PF50: Child Health Questionnaire
CHU9D: Child Health Utility 9D
CRO: clinician-reported outcome
hEDS: hypermobile Ehlers-Danlos Syndrome
G-HSD: Generalised Hypermobility Spectrum Disorder
GJH: Generalised joint hypermobility
Global-VAS: parent’s global assessment
JHS: Joint Hypermobility Syndrome
JIA: Juvenile Idiopathic Arthritis, M-ABC2:Movement Assessment Battery for Children, 2nd Edition NRS:Numerical Rating Scale
PedsQL: Pediatric Quality of life questionnaire
PGIC: Patient’s Global Impression of Change
PODCI: Pediatric Outcomes Data Collection Instrument
PRISMA: Preferred Reporting Items for Systematic Reviews and Meta-Analyses
PRO: patient- or parent-reported outcome
PROSPERO: Prospective Register of Systematic Review
RCT: Randomised controlled trial
VAS: patient-reported Visual Analogue Scale
WBFPS: Wong-Baker Faces Pain Scale
